# Improved Detection of Microsatellite Instability in Early Colorectal Lesions

**DOI:** 10.1371/journal.pone.0132727

**Published:** 2015-08-07

**Authors:** Jeffery W. Bacher, Chelsie K. Sievers, Dawn M. Albrecht, Ian C. Grimes, Jennifer M. Weiss, Kristina A. Matkowskyj, Rashmi M. Agni, Irina Vyazunova, Linda Clipson, Douglas R. Storts, Andrew T. Thliveris, Richard B. Halberg

**Affiliations:** 1 Genetic Analysis Group, Promega Corporation, Madison, Wisconsin, United States of America; 2 Department of Pathology & Laboratory Medicine, University of Wisconsin School of Medicine and Public Health, Madison, Wisconsin, United States of America; 3 Department of Medicine, Division of Gastroenterology and Hepatology, University of Wisconsin School of Medicine and Public Health, Madison, Wisconsin, United States of America; 4 Department of Oncology, University of Wisconsin-Madison, Madison, Wisconsin, United States of America; 5 Department of Ophthalmology and Visual Sciences, University of Wisconsin School of Medicine and Public Health, Madison, Wisconsin, United States of America; University of Naples Federico II, ITALY

## Abstract

Microsatellite instability (MSI) occurs in over 90% of Lynch syndrome cancers and is considered a hallmark of the disease. MSI is an early event in colon tumor development, but screening polyps for MSI remains controversial because of reduced sensitivity compared to more advanced neoplasms. To increase sensitivity, we investigated the use of a novel type of marker consisting of long mononucleotide repeat (LMR) tracts. Adenomas from 160 patients, ranging in age from 29–55 years old, were screened for MSI using the new markers and compared with current marker panels and immunohistochemistry standards. Overall, 15 tumors were scored as MSI-High using the LMRs compared to 9 for the NCI panel and 8 for the MSI Analysis System (Promega). This difference represents at least a 1.7-fold increase in detection of MSI-High lesions over currently available markers. Moreover, the number of MSI-positive markers per sample and the size of allelic changes were significantly greater with the LMRs (p = 0.001), which increased confidence in MSI classification. The overall sensitivity and specificity of the LMR panel for detection of mismatch repair deficient lesions were 100% and 96%, respectively. In comparison, the sensitivity and specificity of the MSI Analysis System were 67% and 100%; and for the NCI panel, 75% and 97%. The difference in sensitivity between the LMR panel and the other panels was statistically significant (p<0.001). The increased sensitivity for detection of MSI-High phenotype in early colorectal lesions with the new LMR markers indicates that MSI screening for the early detection of Lynch syndrome might be feasible.

## Introduction

Lynch syndrome (LS) is a common inherited cancer predisposition characterized by a high risk of developing colorectal, endometrial, gastric, ovarian and other cancers [1]. An estimated 600,000 to 1 million people within the United States have LS, however it is projected that less than 10 percent are currently diagnosed [2]. Methods of screening for LS that are based on family history, age of onset and/or tumor histology suffer from lack of sensitivity and specificity, with diagnosis usually occurring at time of malignancy [3, 4]. Early identification of LS is highly desirable as the risk of developing colorectal cancer can be significantly reduced with increased cancer surveillance [5]. While early tumor development is characteristic of LS, about 60% of colorectal cancer in LS cases may not be diagnosed until after the age of 50 [6]. Thus, screening colorectal lesions obtained during routine colonoscopy that begins around age 50 for evidence of LS could help identify at-risk patients and family members before cancer develops.

Lynch syndrome is caused by germline mutations in one of the DNA mismatch repair (MMR) genes *MLH1*, *MSH2*, *MSH6* and *PMS2* [1]. Loss of MMR activity leads to a high level of mutations, especially in repetitive sequences, referred to as microsatellite instability (MSI). MSI occurs in over 90% of LS cancers and is considered a hallmark of the disease. In 1997, a National Cancer Institute workshop on MSI proposed guidelines for determination of MSI in colon cancer [7]. These guidelines recommended a panel of markers to be used for MSI testing and criteria for MSI tumor classification. In 2004, Revised Bethesda Guidelines for LS suggested use of a panel of all mononucleotide repeats to further increase sensitivity [4].

MSI analysis of colorectal polyps has been reported to be less sensitive than analysis of more advanced neoplasms for detection of LS, therefore, the value of screening polyps remains unclear. Estimates for the incidence of MSI in adenomas range from 41 to 86% (average of 70%), which is comparable to immunohistochemistry (IHC) sensitivity of 49 to 82% (average of 72%) [8–14]. MSI is thought to be a progressive phenomenon in which mutations accumulate over time in a clonal cell population. The MSI phenotype might be milder in early colorectal lesions due to a lower number of cell divisions occurring after loss of MMR activity in comparison to that seen in more advanced neoplasms. However, it is evident that MSI occurs at an early stage of adenoma formation, as it has been found in aberrant crypt foci of microscopic size [15, 16] and has even been observed in normal colonic mucosa of patients with LS [17]. If the sensitivity of screening methods could be increased to levels approaching those seen in colorectal carcinomas, screening adenomas would become a viable option for early identification of LS patients.

To increase sensitivity for detection of MSI, we investigated the use of recently discovered mononucleotide repeats with very long poly-A runs of 40-60bp, which are significantly longer than the mononucleotide repeats that are currently being used for MSI testing [18]. The mutation frequency in mononucleotide repeats increases exponentially with increasing number of repeating units and we speculated that this would translate into increased sensitivity of MSI detection [19–22]. Over 100 long mononucleotide repeats (LMR) markers were identified from BLAST searches of human genome databases, and a subset of markers was selected for further evaluation in this study based on the screening results of 30 mismatch repair deficient endometrial cancers (unpublished data). The goal of this study was to investigate whether the use of these new LMR markers can increase sensitivity for detection of MSI in adenomas to a level approaching that reported in the literature for colorectal carcinomas with current marker systems (i.e., >90%) [23].

## Materials and Methods

### Samples

Patient records from colonoscopies performed between 2003 to 2013 at the University of Wisconsin Hospital and Clinics were reviewed under a protocol approved by the University of Wisconsin-Madison Health Sciences Minimal Risk IRB. No patient consent was required by the IRB as the records were anonymized and de-identified prior to analysis. Selection criteria included patients 55 years of age or less, with one or more adenomas at least 5mm in size and/or with high-grade dysplasia. Patients with a personal or family history of colon cancer were preferentially included. In a few cases where adenoma and carcinoma samples were available, both were analyzed. Archived formalin-fixed paraffin-embedded (FFPE) samples that met our selection criteria were collected.

### Microsatellite instability analysis

DNA was extracted from micro-dissected FFPE tumor sections using Maxwell 16 FFPE Tissue DNA Purification Kit (Promega, Madison, WI) and quantified using a Nanodrop (Thermo Scientific, Wilmington, DE). MSI analysis was performed with three different microsatellite panels: (1) MSI Analysis System (Promega) consisting of mononucleotide repeats BAT-25, BAT-26, NR-21, NR-24, MONO-27 (and pentanucleotide repeats Penta C and Penta D for sample identification) [24], (2) the NCI panel (also known as the Bethesda panel) consisting of mononucleotide repeats BAT-25 and BAT-26, and dinucleotide repeats D2S123, D5S346 and D7S250 [7], and (3) an experimental LMR panel (Promega) containing BAT-52, BAT-55, BAT-56, BAT-57 and BAT-59. The number in the mononucleotide marker name indicates the number of poly-A repeats based on GeneBank GRCh38 reference genome assembly. For detection of MSI, approximately 10 ng of DNA was amplified with 1x primer mix, 1x Gold ST*R Buffer (Promega), and 0.5U GoTaq Hot Start Polymerase (Promega) in a GeneAmp PCR System 9700 Thermocycler (Applied Biosystems, Foster City, CA) following manufacturer’s recommended amplification conditions for the MSI Analysis System. The LMR panel is under commercial development at Promega and long mononucleotide primers will be made available upon request under Material Transfer Agreement. PCR products were denatured in deionized formamide with Internal Lane Standard 600 (Promega) for allele sizing and analyzed on a 3130*xl* Genetic Analyzer using GeneMapper 4.0 Software (Applied Biosystems). Allelic sizes for matching normal and tumor samples were compared and considered MSI unstable if there was a shift of 3bp or more in the tumor allele. Samples were classified as MSI-High (MSI-H) when two or more markers out of a panel of five were unstable, MSI-Low when one out of five markers was unstable and MSI stable when there were no unstable markers.

### Immunohistochemistry analysis

Immunohistochemical analysis was performed using anti-MLH1 (PM220, Biocare Medical, Concord, CA), anti-MSH2 (PM219, Biocare Medical), anti-MSH6 (PM265, Biocare Medical), and anti-PMS2 (PM344, Biocare Medical). Samples were fixed in 10% neutral buffered formalin, embedded in paraffin, and cut into 5 μm sections. Antigen retrieval was performed using 10mM citrate buffer (anti-MSH2) or Tris-EDTA buffer pH 9.0 (anti-MLH1, anti-MSH6, anti-PMS2) heated to boiling for 32 minutes and blocked for endogenous peroxidase with Peroxidazed 1 (PX968, Biocare Medical). Slides were blocked using Background Sniper (BS966, Biocare Medical), then incubated for one hour at room temperature with antibodies diluted 1:100–1:200. Slides were treated with MACH 4 Universal HRP Polymer (M4U534, Biocare Medical), detected with Betazoid DAB Chromogen (BDB2004, Biocare Medical), counterstained with hematoxylin, dehydrated, and cover-slipped. Samples were graded for the absence or presence of nuclear staining of the MMR proteins. Intact staining in the infiltrating immune cells was used as an internal control. A board-certified pathologist who was blinded to the MSI results confirmed all the IHC results.

### BRAF analysis

Samples were screened for BRAF V600E mutations using the amplification refractory mutation system (ARMS)-PCR as described by Huang and colleagues [25] and in two cases confirmed by DNA sequencing using the Ion AmpliSeq Cancer Hotspot Panel v2 (Life Technologies).

### Statistical analysis

The statistical analyses were performed with SigmaPlot (Systat Software, San Jose, CA) to compare differences in the percent of MSI-High lesions detected with individual markers, the number of unstable markers per lesion and the average size shift of MSI-High tumor alleles using the standard t-test and z-test with Yates correction. A *p* value of less than 0.05 was considered to be significant.

The “relative” sensitivity for detection of MSI-High lesions was calculated as the number of lesions classified as MSI-positive for an individual marker divided by the number of MSI-High lesions detected using the NCI panel, the MSI Analysis System or the new LMR panel. The sensitivity and specificity for detection of MMR-deficient lesions was estimated for a subset of 90 samples using IHC expression data for MLH1, MSH2, MSH6 and PMS2 as the gold standard. Sensitivity and specificity for the detection of MSI-High lesions was estimated using the formulas: sensitivity = true positives ÷ (true positives + false negatives) and specificity = true negatives ÷ (true negatives + false positives). True positives were considered MSI-High lesions with loss of MMR expression by IHC (or germline MMR mutation). True negatives were MSI stable lesions with normal MMR expression by IHC. False positive were MSI-High lesions that had normal MMR expression by IHC. False negative were MSI stable lesions with loss of MMR expression by IHC (or germline MMR mutation).

## Results

### Study population and samples

Patients with a history of cancer were preferentially selected for this study resulting in 32.5% (52/160) of cases having a personal or family history of one or more 1^st^ or 2^nd^ degree relatives with colon or other LS-associated cancers. Most were colon cancers (43/52). The other LS-associated cancers were ovarian, uterine and gastric. About six percent (9/160) of patients had a previous colorectal carcinoma and 78% (7/9) of these had synchronous or metachronous lesions in this study. An additional 11% (18/160) had lesions previously removed. Only 3% (5/160) had both a personal and family history of colon cancer. A total of 430 lesions from 160 patients were used in this study. The age at polypectomy ranged from 29 to 55 years, with an average age of 47.5 years. The study population consisted of 54% men and 46% women. Most lesions tested were ≥5mm in size. A board-certified pathologist analyzed each lesion. Of the 414 lesions with clear pathological findings, there were 3 hyperplastic polyps, 287 tubular adenomas, 81 tubulovillus adenomas, 8 villous adenomas, 7 traditional serrated adenomas, 7 sessile serrated adenomas, 2 intramucosal carcinomas, and 19 invasive adenocarcinomas.

### MSI Analysis

**Fig 1:**
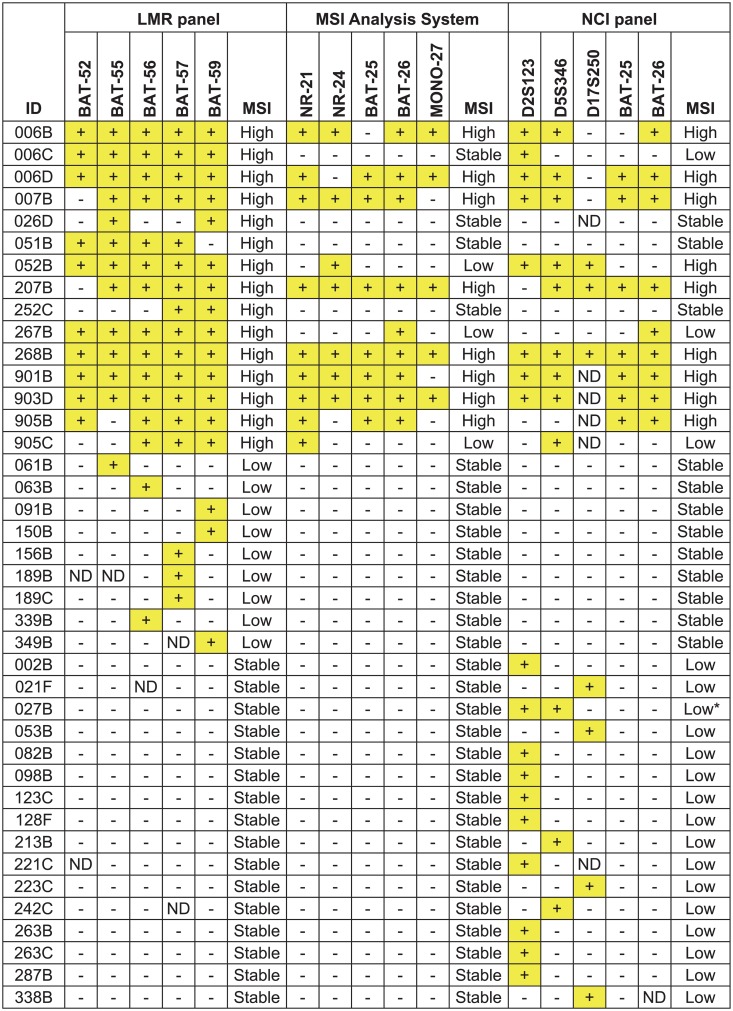
Comparison of MSI results for three marker panels. The MSI classifications of tumor samples are given for all samples that were scored for all three panels and positive for at least one marker from the experimental LMR panel (Promega), the MSI Analysis System (Promega) or the NCI panel. Samples were classified as MSI-High when two or more markers out of a panel of five were unstable, MSI-Low when one out of five markers was unstable and MSI stable when there were no unstable markers. The MSI stable samples with intact MMR staining are not shown. Sample 011C is not shown owing to inconclusive results with the LMR panel. *Sample 027B was scored as MSI-Low for the NCI panel because only the dinucleotides were unstable. Tumors in which only dinucleotides are unstable are often misclassified as MSI-High and typically show MMR expression by IHC, as was the case for sample 027B [7, 49]. +, MSI-positive;-, MSI stable; ND, not done.

To determine whether the detection of MSI in early colorectal lesions could be increased using LMR markers, 430 lesions were screened using the MSI Analysis System, the NCI panel, and an experimental LMR panel (results for all samples that were scored for all three panels and positive for at least one marker are shown in Fig 1). In the majority of patients (127/160), matching normal tissue was available for comparison. In the cases without matching normal tissue, we were still able to determine MSI status of tumors using the MSI Analysis System, because it uses markers that are nearly monomorphic [24]. This characteristic allows MSI analysis to be performed in the absence of matching normal tissue with over 95% accuracy by comparing the tumor allele size with the common allele size in the population [26]. The dinucleotide repeats in the NCI panel and the LMRs are polymorphic and matching normal tissue is usually needed for MSI determination. However, MSI determination is still possible with polymorphic markers without matching normal tissue in some circumstances. For example, any tumor sample that had three or more alleles per marker was considered to be MSI-positive, as this is a rare event in normal cells. Also, in cases where three or more lesions were available from the same individual, the common allelic pattern was used as a surrogate for an individual’s “normal” genotype.

**Fig 2:**
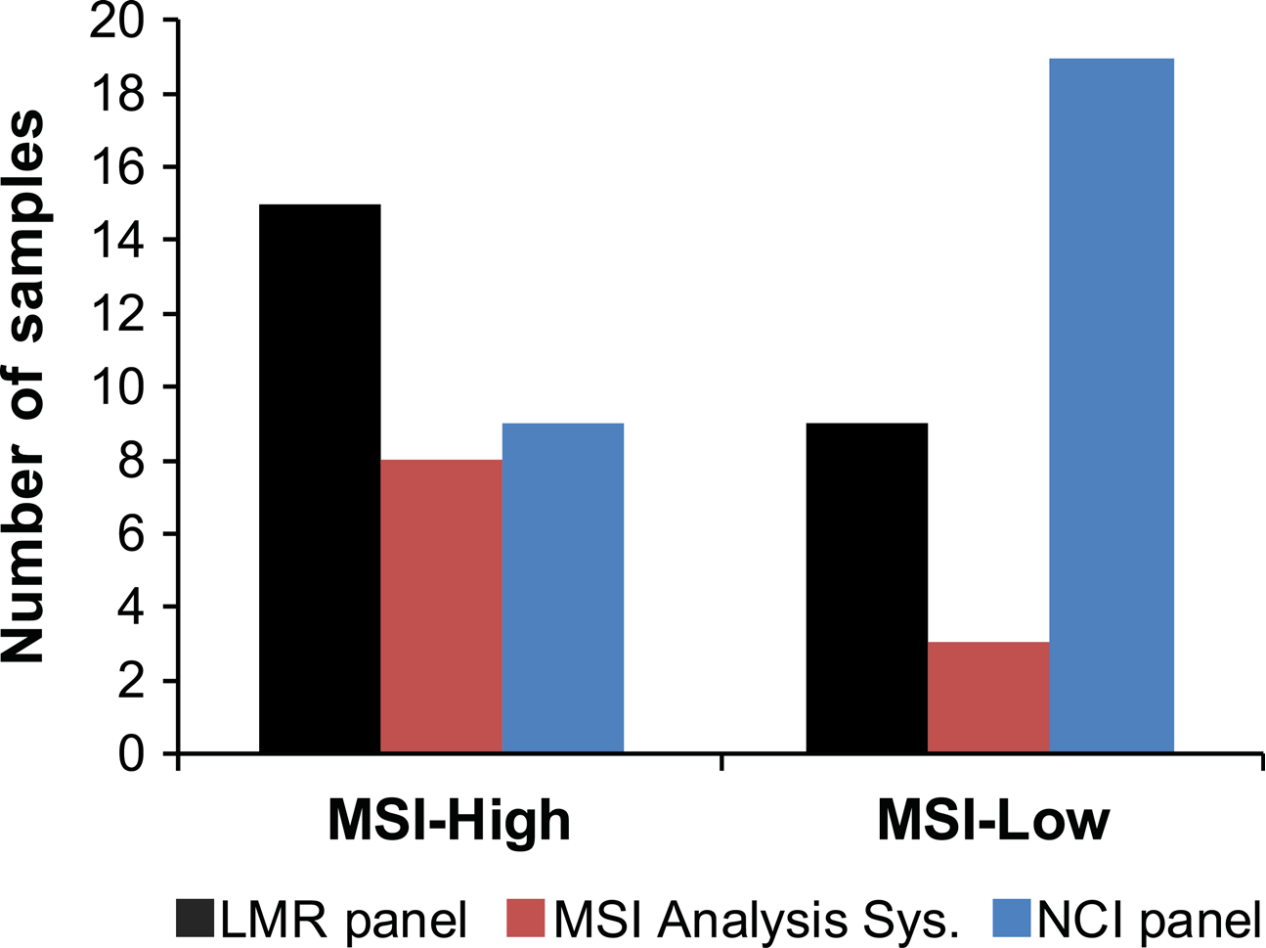
Use of LMR markers increased detection of MSI-High colonic lesions by 1.7 to 1.9 fold over currently used MSI markers. Three marker panels were used to screen lesions from 160 patients for MSI, including the experimental LMR panel, the MSI Analysis System and the NCI panel and the. Samples with two or more markers out of five unstable were considered MSI-High and one marker out of five as MSI-Low.

**Fig 3:**
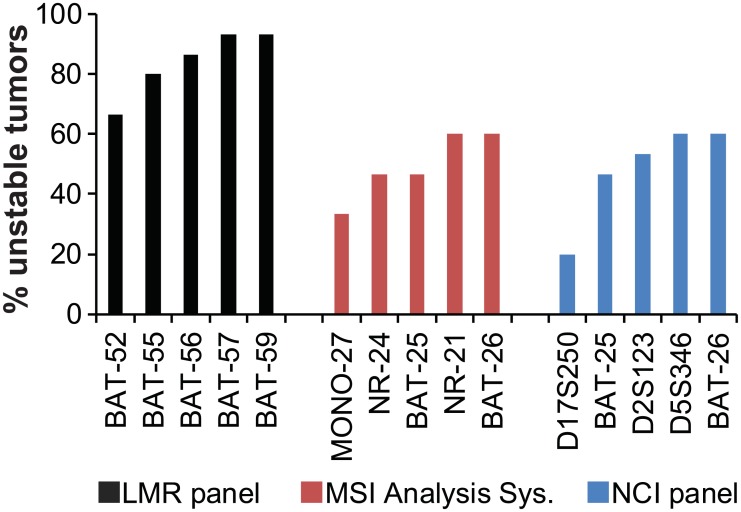
The relative MSI sensitivity of the LMR markers was significantly higher than that of currently used markers. A total of 15 tumors were classified as MSI-High using the LMR panel, the MSI Analysis System or the NCI panel. The percentage of tumors that were scored as MSI-positive is shown for each individual marker. The relative sensitivity of the LMR panel was significantly higher than that of the MSI Analysis System (p = 0.0012) and the NCI panel (p<0.0038) using the t-test.

Overall, 15 tumors were scored as MSI-High using the LMR panel compared to 9 for the NCI panel and 8 for the MSI Analysis System (Fig 2). This represents a 1.7 to 1.9-fold increase in detection of MSI-High lesions over currently used markers. The relative MSI sensitivity of individual LMR markers varied, but even the worst marker was more sensitive than the markers in the NCI panel and the MSI Analysis System (Fig 3). The sensitivity and specificity for detection of MMR-deficient lesions was estimated for a subset of 90 samples for which there was MMR expression data by IHC (Table 1). The overall sensitivity and specificity of the LMR panel for detection of MMR-deficient lesions was 100% and 96%, respectively. In comparison, the sensitivity and specificity of the MSI Analysis System was 67% and 100%; and for the NCI panel, 75% and 97%. The difference in sensitivity between the LMR panel and the both the MSI Analysis System and the NCI panel was statistically significant (z-test; p<0.001). The specificity of the LMR panel was not significantly different from the MSI Analysis System or the NCI panel (z-test; p = 0.252 and 0.899, respectively). Limitations for these estimates are that IHC testing was not performed on all samples and that in most cases the germline MMR mutation status was unknown. Therefore, the values may be overestimates and only refer to detection of mismatch-deficient lesions, not to detection of Lynch syndrome.

**Table 1:** Estimated sensitivity and specificity of panels and markers for detection of MMR-deficient lesions.

Panel/marker	True positive	False negative	True negative	False positive	Sensitivity (%)	Specificity (%)
LMR panel	12	0	67	3	100	96
BAT-59	12	0	70	4	100	95
BAT-57	12	0	63	3	100	95
BAT-56	12	0	69	2	100	97
BAT-55	10	2	68	5	83	93
BAT-52	10	2	70	1	83	99
MSI Analysis System	8	4	75	0	67	100
BAT-26	9	3	75	0	75	100
NR-21	9	3	75	0	75	100
NR-24	7	5	75	0	58	100
BAT-25	7	5	75	0	58	100
MONO-27	6	6	75	0	50	100
NCI panel	9	3	66	2	75	97
BAT-26	9	3	75	0	75	100
D2S123	9	3	65	5	75	93
BAT-25	7	5	75	0	58	100
D17S250	2	6	69	1	25	99
D5S346	8	4	68	3	67	96

The MMR status of 90 lesions was determined by IHC for MLH1, MSH2, PMS2 and MSH6. MSI data was not available in all lesions; therefore the result categories do not sum to 90. There were 12 lesions that did not stain for one or more MMR proteins and these were considered true positives for loss of MMR. There were 78 lesions that stained normally for all MMR proteins and were considered true negatives with normal MMR expression. For panels, a result of MSI-High was considered positive, and a result of MSI-Low or MSI stable was considered negative. The difference in sensitivity between the LMR panel and the both the MSI Analysis System and the NCI panel was statistically significant (z-test; p<0.001). The specificity of the LMR panel was not significantly different from the MSI Analysis System or the NCI panel (z-test; p = 0.252 and 0.899, respectively).

A low level of MSI was observed in some lesions for all three panels. The number of MSI-Low samples observed with the LMR panel was intermediate between the NCI panel and the MSI Analysis System (Fig 2). A higher incidence of MSI-Low cases with the NCI panel can be attributed to the lower specificity of dinucleotide repeats for detection of MMR loss [4, 24]. In contrast, the lower incidence of MSI-Low with the MSI Analysis System can be attributed to high specificity exhibited by the mononucleotide repeats in this panel [24]. The samples scored as MSI-Low with the MSI Analysis System were all MMR-deficient, but were considered false negatives as samples were grouped into MSI-High and “not MSI-High” categories for this study. The importance of MSI-Low classification is controversial as the occurrence of a MSI in a single marker could be due to background mutations and is not generally associated with loss of MMR function [27]. In comparison, the MSI-Low cases detected with the LMR and NCI panels showed intact MMR expression and were mutually exclusive, indicating that they were unlikely to be the result of MMR defects.

**Fig 4:**
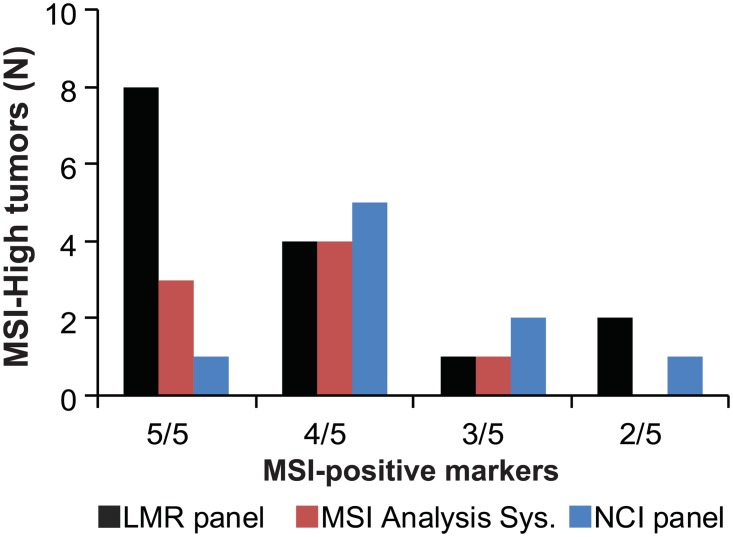
The number of MSI-positive markers per sample was highest for the LMR panel and increased confidence in MSI classification. The number of markers that were MSI-positive for each of 15 MSI-High samples is given for the three marker panels. The number of unstable markers per sample was significantly higher for the LMR panel compared to the other panels (z-test; p<0.001). MSI analysis with LMRs resulted in MSI-High samples with either 5/5 or 4/5 unstable markers in 80% of cases.

**Fig 5:**
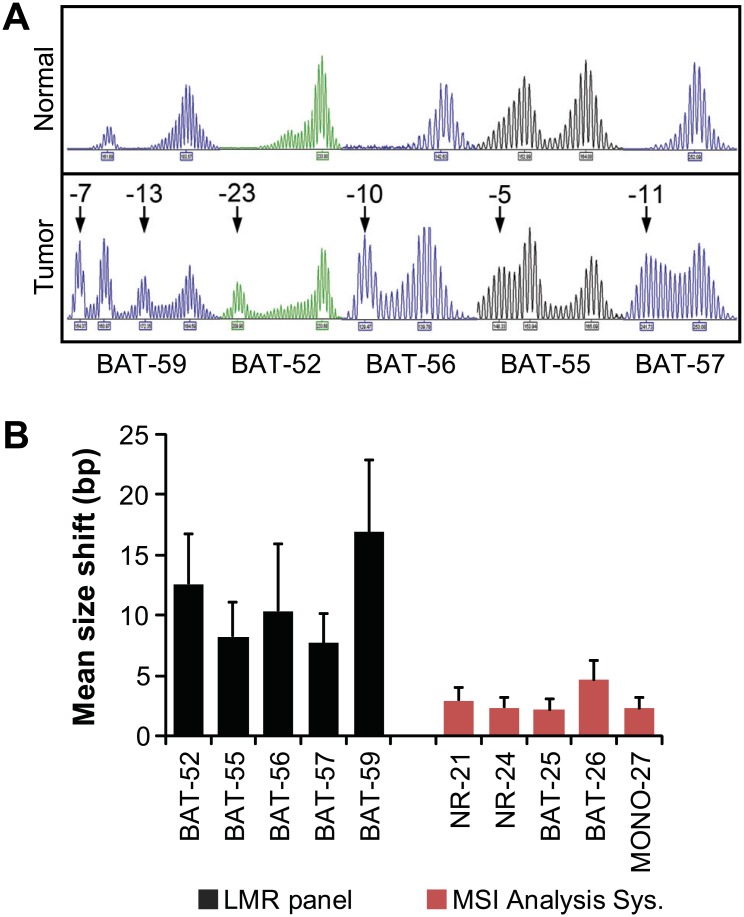
Use of LMR markers for MSI analysis typically resulted in larger allele size changes in MSI-High lesions. (A) Electropherogram of MSH2-deficient tumor sample 267B and matching normal tissue screened for MSI using the experimental LMR panel which shows all five markers were unstable with size shifts of up to 24bp. (B) The average change in tumor allele size of MSI-High lesions was determined for the markers in the LMR panel and the MSI Analysis System. The average size shift was significantly greater in LMR markers (p = 0.0014), which increased confidence in MSI classification. Error bars show standard error of the mean.

In addition to an overall increase in MSI frequency, the number of MSI-positive markers per sample and the size of allelic changes were also greater with the LMR panel. The significantly higher number of LMR markers that were MSI-positive for a given sample increased confidence in MSI classification (Fig 4). MSI analysis with the LMR panel resulted in MSI-High samples with 5/5 or 4/5 unstable markers in 80% of cases. With one exception, these exhibited loss of MMR expression by IHC or had germline MMR mutations (Table 2). The significantly larger size changes in LMRs simplified MSI classification by reducing the number of ambiguous calls associated with small allele size changes (Fig 5). Very small size changes of one to two base pairs were commonly observed in all markers and were considered to be due to normal assay variation reflecting slippage of the polymerase. To minimize this noise, only size shifts of 3bp or more were scored as MSI-positive.

**Table 2:** Characteristics of the 15 samples classified as MSI-High by the LMR panel from 9 patients

Patient	Personal History^a^	Family History^b^	Sample	Pathology^c^	Tumor Location	LMR Panel	MSI Analysis System	NCI Panel	IHC or mutation^d^	BRAF V600E^e^	Group^f^
1	No	CRC-73	901B	TA	Right	5/5	5/5	4/4	MLH1	No	LS
2	CRC-55	CRC-46	903D	AC	Right	5/5	5/5	4/4	MSH2	No	LS
3	CRC-49	EC-late 50s	905B	AC	Right	4/5	3/5	2/4	MSH6	No	LS
			905C	TA	Left	3/5	1/5	1/4	MSH6	No	
4	CRC-50	CRC–40	006B	TA	Left	5/5	4/5	3/5	MLH1	No	LS probable
			006C	TA	Left	5/5	0/5	1/5	MLH1	No	
			006D	TA	Left	5/5	4/5	4/5	MLH1	No	
			007B	AC	Right	5/5	4/5	4/5	MLH1	No	
5	No	CRC-34	051B	HP	Left	5/5	0/5	0/5	Normal	No	LS probable
			052B	TA	Left	5/5	1/5	3/5	MSH2	No	
6	CRC-49	No	207B	AC	Right	5/5	4/5	3/5	MLH1	No	LS probable
7	No	OV-mid 40s; CRC-?	267B	TA	Right	5/5	1/5	1/5	MSH2	No	LS probable
			268B	TVA	Left	5/5	5/5	5/5	MSH2	No	
8	No	No	026D	TA	Left	2/5	0/5	0/4	Normal	No	Unknown
9	No	No	252C	SSP	Right	2/5	0/5	0/5	Normal	No	Unknown

^a^ Personal history of LS-associated cancer. CRC,colorectal cancer; number indicates age in years at diagnosis.

^b^ 1^st^ or 2^nd^ degree relative with LS-associated cancer. CRC,colorectal cancer; OV,ovarian cancer; EC,endometrial cancer; number indicates age in years at diagnosis; question mark indicates unknown age.

^c^ AC, adenocarcinoma; HP, hyperplastic polyp; SSP, sessile serrated polyp; TA, tubular adenoma; TVA, tubulovillous adenoma.

^d^ ND, no data. MLH1, loss of MLH1 and PMS2 expression by IHC. MSH2, loss of MSH2 and MSH6 expression by IHC. Normal, intact IHC staining. Germline DNA sequencing information was available for patients 1–3.

^e^ BRAF V600E mutation tested by DNA sequencing and/or by ARMS-PCR [25].

^f^ Group: Patients were classified as LS if they had a pathogenic germline MMR mutation. Patients were classified as LS probable if tumor was MSI-High and no BRAF V600E mutation was found, plus one of more of the following: loss of expression for MSH2, MSH6 or PMS2 by IHC, colorectal cancer before age 50 or a 1^st^ or 2^nd^ degree relative with LS-associated cancer.

Detection of MSI in cancers with *MSH6* mutations can be problematic as they often exhibit an attenuated MSI phenotype, as well as reduced penetrance of colorectal cancer and a later age of onset [28]. This challenge is especially relevant when using the NCI panel, which contains dinucleotide repeats that are not as sensitive to MSI induced by *MSH6* loss. There were two samples (905B and 905C) with *MSH6* germline mutations. Sample 905B was an adenocarcinoma and was MSI-High with all three marker panels (Fig 1). 905C was a synchronous tubular adenoma from the same patient and was classified as MSI-High only with the LMR panel.

### Immunohistochemistry and BRAF analysis

**Fig 6:**
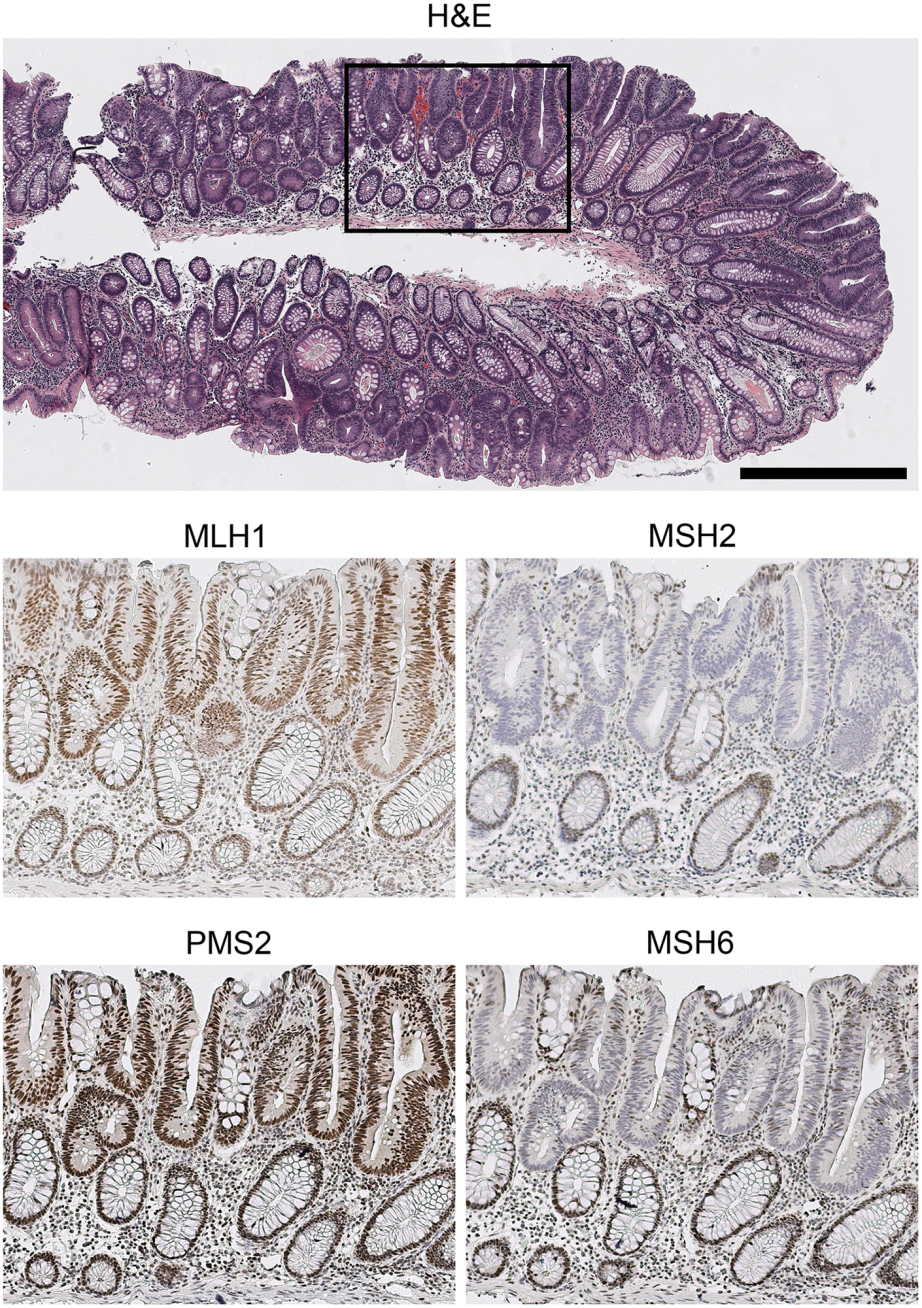
High concordance between IHC and MSI results using LMRs. There was 96% (79/82) concordance between MSI results using LMR repeats and loss of MMR expression by IHC. For example, tubular adenoma 267B was unstable at all five markers and lacked MSH2 and MSH6 expression. Note that when MSH2 is lost, the level of binding partner MSH6 is often significantly lower due to reduced stability. The area indicated by the rectangle in the H&E panel is enlarged 2x in each of the lower panels. Size bar for H&E, 500μm.

Immunohistochemical analysis for MMR protein expression for MLH1, MSH2, PMS2 and MSH6 was performed on a subset of 90 lesions. In addition, germline sequencing data was available for three patients with MSI-High tumors for which loss of tumor MMR expression was also confirmed by IHC. Out of the 15 MSI-High samples identified using the LMR panel, 12 exhibited loss of MMR expression or had germline mutations (Table 2 and Fig 6). Conversely, 100% of samples showing loss of MMR expression were MSI-High with the LMR panel. All tumors classified as MSI stable or MSI-Low with the LMR panel exhibited normal MMR expression. National Comprehensive Cancer Network (NCCN) guidelines recommend BRAF testing to distinguish between sporadic MLH1 deficient tumors caused by promoter methylation with associated BRAF mutations and loss of MLH1 expression in Lynch syndrome tumors by germline mutation and absence of BRAF mutations [29]. Thus, lack of BRAF V600E mutations in the MSI-High tumors in this study is consistent with LS (Table 2).

The concordance between IHC and MSI results using the LMR panel was 96% (79/82). Three of the MSI-High cases (051B, 026D, 252C) exhibited intact IHC staining and therefore were potentially false negatives that would have been missed if only IHC had been performed (Table 2). For example, patient 5 exhibited MSI at 5/5 makers in hyperplastic polyp 051B, had a tubular adenoma at age 35 with loss of MSH2 expression, a family member with colorectal carcinoma at age 34, was negative for BRAF V600E mutation, and therefore probable LS. The other two samples retaining MMR expression exhibited MSI at 2/5 markers, lacked BRAF V600E mutations, but had no history of colorectal cancer. Therefore, the status of these cases remains unclear, but we cannot rule out the possibility that they are LS. This means that at least 78% (7/9 patients) MSI-High lesions detected with the LMR panel were LS (based on germline MMR mutations) or probable LS (based MSI-High tumor phenotype, loss of MMR expression by IHC, lack of BRAF V600E mutation, early age of onset and cancer history). This high rate of specificity for LS is surprising in light of the fact that around 80% of MSI-High colorectal carcinomas are sporadic (i.e., of the ~15% of colorectal cancers that are MSI-High, only 1 in 5, or 20%, are LS) [1].

The concordance between IHC and MSI results was 95% (83/87) for the MSI Analysis System and 94% (75/80) for the NCI panel. The four discordant cases with the MSI Analysis System were 006C, 052B, 267B and 905C (Table 2). These were scored as MSI stable or MSI-Low, but exhibited loss of MMR expression by IHC and were therefore likely false negatives. There were five discordant IHC cases with the NCI panel. Three of these were the same samples misclassified with the MSI Analysis System (006C, 267B, 905C). The other discordant samples with the NCI panel were 011C and 027B, each of which exhibited MSI in 2/5 markers, both dinucleotides. Misclassification of tumors as MSI-High has been reported with the NCI panel when two dinucleotide markers are unstable in the absence of instability in mononucleotides [4, 24]. 011C was MSI stable with the MSI Analysis panel and 027B was MSI stable with the LMR and MSI Analysis panels. Moreover, both of these samples retained MMR expression. Therefore, 011C and 027B are likely false positives.

### Clinicopathologic characteristics of MSI-High samples

The characteristics of the 15 samples classified as MSI-High by the LMR panel from 9 patients are summarized in Table 2. Of these samples, 8 were tubular adenomas, 1 tubulovillous adenoma, 1 sessile serrated polyp, 1 hyperplastic polyp and 4 adenocarcinomas. Left (n = 8) and right (n = 7)-sided MSI-High tumors occurred with similar frequencies; however, all four carcinomas were right-sided. We detected MSI-High tumors in 12% (6/52) of patients with a family history of LS-associated cancers, all of which we classified as LS or probable LS.

A history of MSI-High colorectal adenomas was also associated with an increased risk of the occurrence of additional MSI-High adenomas and carcinomas. For example, patient 4 had three MSI-High tubular adenomas removed at age 49 and one year later a MSI-High adenocarcinoma (Table 2). Patient 5 had a MSI-High hyperplastic polyp removed at age 29 and a MSI-High tubular adenoma at age 35. Patient 7 had a MSI-High tubulovillous adenoma removed at age 37 and a MSI-High tubular adenoma at age 39. Finally, patient 3 had a synchronous MSI-High tubular adenoma and MSI-High adenocarcinoma at age 49. All cases in which two or more lesions were MSI-High were classified as either LS or probable LS.

## Discussion

A large number of colonic adenomas are removed during routine colonoscopies and are found in roughly one third of asymptomatic individuals between the ages of 50 and 75 [30]. Since MMR deficiency is an early event in colorectal tumorigenesis, screening adenomas for MSI could be a useful strategy for early detection of LS. Analysis of previous studies on detection of MMR deficiency in LS adenomas showed an average sensitivity of approximately 70% [8–14, 31, 32]. In comparison, the sensitivity of MSI testing in LS colorectal carcinomas is over 90% [1]. In an effort to increase the MSI sensitivity in pre-cancerous lesions, we tested a new panel of LMRs that have been shown to increase sensitivity [19, 33]. Overall, 15 of the tested tumors were scored as MSI-High using the LMR panel compared to 9 for the NCI panel and 8 for the MSI Analysis System. This difference represents a 1.7 to 1.9 fold increase in detection of MSI-High lesions over currently used markers. In addition, the number of markers that were MSI-positive per sample and the size of allelic changes were significantly greater with the LMR panel, which greatly increased confidence in MSI classification (Figs 4 and 5).

One of the more interesting findings from this study was the high proportion of MSI-High adenomas in likely LS cases. Approximately 80% of MSI-High colorectal cancers in the general population are spontaneous cancers caused by somatic inactivation of MMR via promoter methylation. Yet MSI-High adenomas in this study appear to be predominately associated with LS. This high correlation between MSI-High adenomas and LS has been reported, but the reason is still unknown [12, 34]. One possible explanation for the apparent lack of MSI-High adenomas observed in non-LS patients is that sporadic MSI-High tumors arise primarily through the serrated pathway while LS tumors arise from traditional adenomas (tubular, tubulovillus and villus adenomas) [35, 36]. Lesions collected for this study consisted predominantly of traditional adenomas and this may have enriched for LS. It is also possible that MSI occurs earlier in tumor development in LS individuals than in sporadic cases and therefore is more likely to be detected in adenomas of patients with LS. Whatever the cause, the detection of MSI in pre-cancerous lesions might be a useful indicator of rapid tumor progression with direct implications on colonoscopy intervals. This possibility was illustrated in one of the patients in this study who had an adenoma removed and one year later developed an adenocarcinoma.

MSI is predominately seen in proximal or right-sided colorectal cancers. This is especially true in sporadic MSI-High cancers where over 90% may be located in the proximal colon [37, 38]. However, right-sided location is less prevalent in LS carcinomas (20–62%) [39, 40] and adenomas (27–51%) [8, 13, 14, 41]. The apparent higher incidence of right-sided carcinomas may be due in part to the more rapid progression of tumors in the proximal colon [41]. In our study, left- and right-sided MSI-High tumors occurred with similar frequencies; however, all four carcinomas were right-sided (Table 2). Based on these findings, it would appear that using right-sided location as a selection criteria for screening adenomas would result in missing a substantial proportion of LS tumors.

The optimal strategy for identifying individuals with LS is a subject of continued debate. Some advocate universal screening of all colorectal carcinomas, while others prefer targeted screening based on age of onset, family history and/or histologic criteria to reduce the number of unnecessary tests [3, 4, 6, 42, 43]. For example, Moreira and colleagues compared various strategies for identifying patients with LS and found that the revised Bethesda guidelines had a sensitivity of 87.8% compared with 100% sensitivity of the universal screening approach [43]. These strategies all involve testing carcinomas and therefore miss the opportunity to detect LS before cancer develops. However, if MSI screening of adenomas was sufficiently sensitive for detection of LS this approach would provide an additional benefit of being able to identify at-risk LS individuals and family members before cancer develops.

Use of MSI or IHC as the primary screening method for detection of LS is also a subject of ongoing debate. IHC analysis is a valuable tool, but many believe that PCR-based MSI analysis is currently indispensable and cannot be replaced by IHC alone. The NCCN guidelines state that there is a 5% to 10% false negative rate for IHC [29]. False negative IHC results may be caused by retained epitopes in non-functional proteins that may still be antigenically detectable or due to various technical and interpretive problems [4, 6, 24, 44–46]. The significance, use and implications for MSI testing are similar to those for IHC, although the tests are slightly complementary. In general, MSI test results are relatively easy to interpret and highly reproducible between observers. MSI is also a functional test that can help determine clinical significance of variants of unknown significance, which account for up to one third of MMR gene mutations [47]. The InSiGHT international database that collects MMR gene variants already lists 2,360 variants. [48]. Such variants present an interpretive challenge and can cause counseling dilemmas related to the understanding and psychological impact of uncertain test results. Determining tumor MSI status also provides prognostic and therapeutic value for individualizing treatment not only for LS patients, but also for those with MSI-High sporadic colorectal cancer [3].

MSI testing of polyps has been proposed for the early detection of LS, but is currently not a realistic, cost-effective approach due to low sensitivity in early pre-cancerous lesions. The goal of this study was to investigate whether the use of new LMR markers can increase detection of MSI in adenomas to a level approaching that reported for colorectal carcinomas with current marker systems (i.e., >90% sensitivity) [23]. The use of the LMR panel did significantly increase the sensitivity (100% for this study population) for detection of mismatch repair deficient lesions over currently available marker panels without significantly decreasing specificity. Studies to determine the sensitivity for detection of LS still need to be performed to determine if the higher levels of MSI achieved using the new LMR markers will transform MSI testing into a practical screening tool for pre-cancerous lesions and early detection of LS.

## Supporting Information

S1 Dataset Supplementary Data. (XLSX)
